# Metagenomic analysis of soil and freshwater from zoo agricultural area with organic fertilization

**DOI:** 10.1371/journal.pone.0190178

**Published:** 2017-12-21

**Authors:** Aylan K. Meneghine, Shaun Nielsen, Alessandro M. Varani, Torsten Thomas, Lucia Maria Carareto Alves

**Affiliations:** 1 Department of Technology, São Paulo State University (Unesp), School of Agricultural and Veterinarian Sciences, Jaboticabal, São Paulo State, Brazil; 2 Centre for Marine Bio-Innovation, University of New South Wales, Sydney, New South Wales, Australia; 3 School of Biological, Earth and Environmental Science, University of New South Wales, Sydney, New South Wales, Australia; Agroecological Institute, CHINA

## Abstract

Microbial communities drive biogeochemical cycles in agricultural areas by decomposing organic materials and converting essential nutrients. Organic amendments improve soil quality by increasing the load of essential nutrients and enhancing the productivity. Additionally, fresh water used for irrigation can affect soil quality of agricultural soils, mainly due to the presence of microbial contaminants and pathogens. In this study, we investigated how microbial communities in irrigation water might contribute to the microbial diversity and function of soil. Whole-metagenomic sequencing approaches were used to investigate the taxonomic and the functional profiles of microbial communities present in fresh water used for irrigation, and in soil from a vegetable crop, which received fertilization with organic compost made from animal carcasses. The taxonomic analysis revealed that the most abundant genera were *Polynucleobacter* (~8% relative abundance) and *Bacillus* (~10%) in fresh water and soil from the vegetable crop, respectively. Low abundance (0.38%) of cyanobacterial groups were identified. Based on functional gene prediction, denitrification appears to be an important process in the soil community analysed here. Conversely, genes for nitrogen fixation were abundant in freshwater, indicating that the N-fixation plays a crucial role in this particular ecosystem. Moreover, pathogenicity islands, antibiotic resistance and potential virulence related genes were identified in both samples, but no toxigenic genes were detected. This study provides a better understanding of the community structure of an area under strong agricultural activity with regular irrigation and fertilization with an organic compost made from animal carcasses. Additionally, the use of a metagenomic approach to investigate fresh water quality proved to be a relevant method to evaluate its use in an agricultural ecosystem.

## Introduction

Microbial communities are diverse and complex, playing an important role in organic matter decomposition and nutrient cycling. Knowledge of microorganisms involved in these processes is important to the understanding of biogeochemical cycles and how they maintain the biosphere [1]. Among the cycles, nitrogen is one of the most abundant and important cycling elements on Earth, it influences other global element cycles and has a critical role in primary production in the majority of the ecosystems [2–4]. In agriculture, the nitrogen supply has been increasingly utilized for sustainable food production, because of that the use of alternative fertilizers such as organic compost and the exploitation of beneficial microorganisms has increased [4,5].

With metagenomic approaches it is possible to investigate and unravel the gene functions and microorganisms involved in biogeochemical cycle in various ecosystems. Metagenomic approaches have been used to understand and describe taxonomic and functional diversity in several environments, such as sediment, organic compost, and agricultural soils as well as water from rivers and oceans [6–8].

Agricultural soils are areas of intense anthropogenic activity, which principally aims at improving soil quality and plant productivity [9,10]. Application of organic amendments, such as compost, has been successful in many cases in improving soil quality and providing an efficient and economic way to stabilize and recycle agricultural biomass [6,10,11]. Many different biomass residues can be used for compost, such as vegetable, sewage, animal manure and carcasses [12–14]. The use of carcasses in organic composts has raised concerns about the potential presence of pathogens, however heat production during composting might mitigate this by killing mesophilic pathogens [15].

Previous microbial community studies on agricultural soils treated with different organic amendments showed an increase of bacterial diversity due to the nutrient enrichment provided by the organic fertilizer, regardless of the site or climate [9,13,16]. By comparing the use of chemical fertilizers (based on nitrogen, phosphorus and potassium forms) and cow manure-based compost, Chaudhry et al [17] demonstrated that organic amendment can enhance the bacterial population of certain phyla which can be correlated with other soil properties such soil organic carbon and nitrogen. However, despite the many studies on soil microbial communities under different organic amendments, little information is available regarding long-term fertilization with an organic compost made of different carbon sources, including animal carcass.

Soils can also be influenced by the irrigation water used, which, if polluted with high nutrient concentrations, toxic elements or pathogens, can negatively affect the environment and food produced in an agricultural area [18,19]. This makes water quality diagnostics extremely important for addressing public health concerns [20,21]. Studies on tracking potential pathogen diversity and abundance though a next-generation sequencing (NGS) approach have increased [20–23]. Employment of metagenomic based-methods to screen for harmful cyanobacterial blooms, contaminant biodegradation, pathogens and their functional genes, allows identification of potential risk agents, semi-quantitative inference on their relative abundance and generic genetic capabilities of microorganisms on aquatic environments [23–26]. Also, there is still a lack of information on how microbiological quality of irrigation freshwater influences the safety of soils and vegetables produced, especially in Brazil. The application of organic fertilizers and the irrigation water are thus key factors that determine soil quality and productivity.

In this work, we investigated the microbiome and the functional gene profile of soil from a vegetable field and the fresh water used for crop irrigation in an agricultural area of the São Paulo Zoo farm park in Brazil. Our main questions were regarding the assessment of fresh water quality, and the comparison of samples at a taxonomic level given the proximity of the sites and the shallow water stream of this peculiar agricultural area. In this area, over the last ten years, there has been an unusual fertilization treatment, which involved organic compost made from several vegetable and animal residues, including carcasses. A previous study showed that the organic compost applied to this soil has an abundance of bacterial genus *Lactobacillus* and genes encoding proteins related to pectin degradation [6]. The effect of this particular organic compost on the soil had never been studied and neither has the quality of the water used for crop irrigation.

## Materials and methods

### Study area and sampling

**Fig 1:**
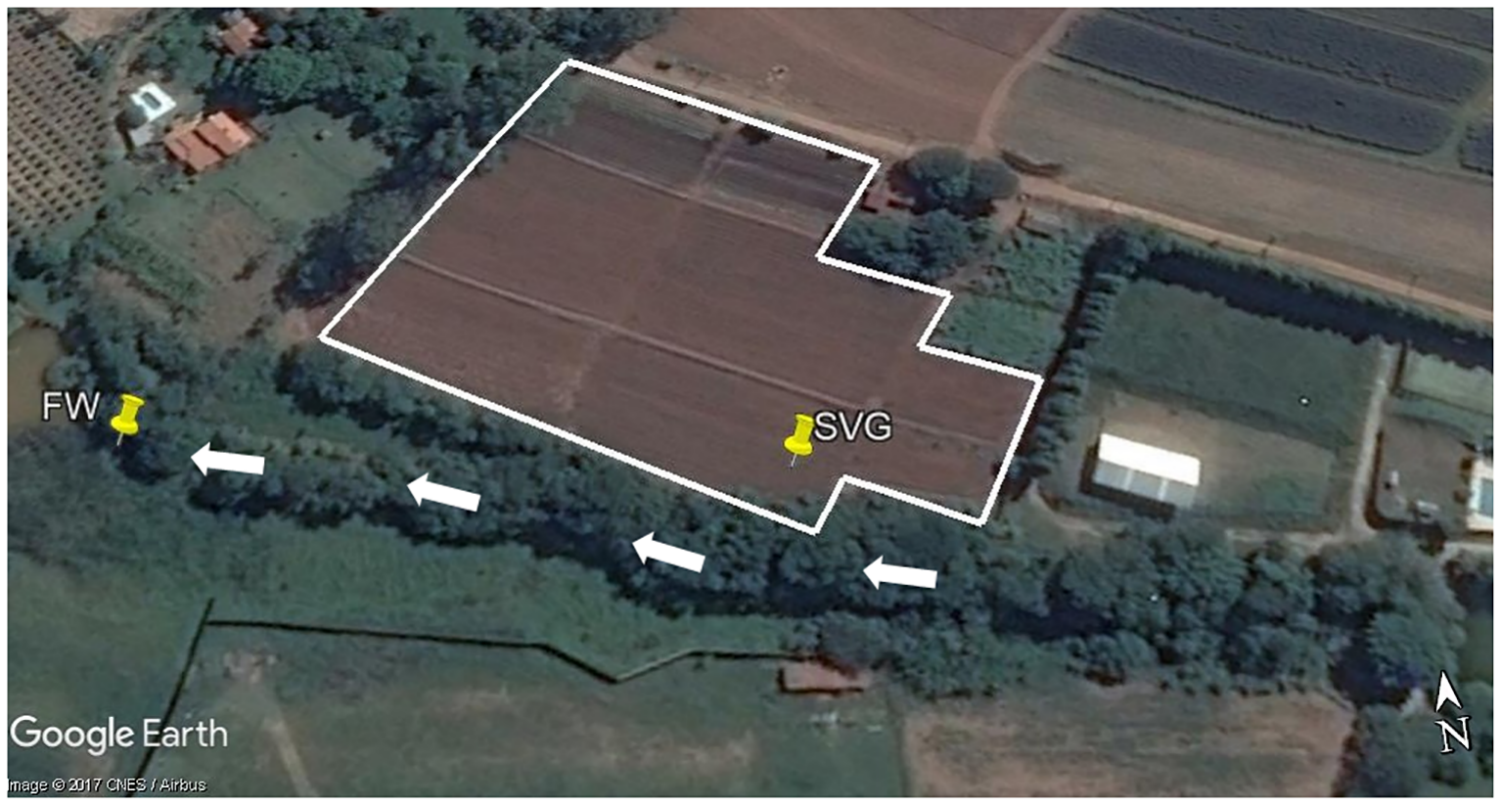
Image of the studied area at São Paulo Zoo Park farm geographical location. SVG is the geographical coordinate of the soil from vegetable crops (23°34′47.17"S; 47°35′12.02"W) and FW is the geographical coordinate of the fresh water used for crop irrigation (23°34’46.07”S; 47°35’17.46”W). The area highlighted in white is SVG which has an area of about 1.5 hectare and is divided into beds of 7 m x 2 m, containing planted carrots, cabbage, lettuce, chicory and beet. The white arrows represent the direction of fresh water flow along the stream.

The study was carried out in the São Paulo Zoo Park farm (FPZSP), located in Araçoiaba da Serra–São Paulo, Brazil [27] (Fig 1). Since 2005, the park farm has applied an organic compost made from vegetable and animal residues, including carcasses from small and large animals [6]. The organic compost has been applied at a rate of 200 t ha^-1^ to the agricultural site that produces vegetables for animal feed of the zoo. Furthermore, mineral fertilizers (superphosphate, potash, borax and ammonium sulphate) have been applied to the vegetable crop, with the purpose of fulfilling specific nutritional requirements of the crops planted.

The vegetable crop is irrigated from a fresh water stream (Ribeirão do Lajeado) located 30 m from the area of vegetable cultivation. The stream has an average elevation of 637 m and belongs to the basin area of the Sorocaba and Medium Tietê rivers. This stream is under the influence of agricultural activities carried out at the farm park, as well as agricultural activities conducted by rural properties upstream.

Sampling was performed in September 2014, a period in which the average temperature was about 22°C and the rainfall was 12 mm (http://www.inmet.gov.br/portal/). In this period, the season was characterized as dry, however, for the two days preceding the sample collection of the samples, there was a light rain in the region. Three days before sampling, mineral fertilizer was applied to the soil of the vegetable crop.

The soil from vegetable crops (SVG) and fresh water used for crop irrigation (FW) located in the same agricultural area were the basis of this study. Three soil samples (0–20 cm depth) with a distance about 50 m from each other, without roots, were collected randomly within the vegetable crop. Fresh water samples (0–30 cm depth) were also collected in triplicate, adjacent to a pump tap that collects stream water for crop irrigation. The water collection point was located at the bottom of the vegetable crop. Each sample was analysed separately.

### Physical and chemical analysis of soil and fresh water

Soil samples were air dried and gently disaggregated. Soil organic matter (OM), elemental analysis (Ca, Cu, Mg, Mn, P, K, Al, Fe, Zn) and pH were measured according to the methods described by Van Raij et al. [28].

For the fresh water samples, measurements of electrical conductivity (EC), dissolved oxygen (DO), pH, oxidation-reduction potential (ORP), total dissolved solids (TDS) and turbidity were determined on site, with a Horiba U-50 multiparameter probe. The chemical parameters ammoniacal nitrogen (N-NH_4_^+^, phenate method); nitrate (N-NO_3_^-^, reduction method), nitrite (N-NO_2_^-^, colorimetric method using NED dihydrochloride), total phosphorus (TP, digestion method with sulfuric and nitric acids) and dissolved reactive phosphorus (DRP, Ascorbic acid method) were determined by molecular spectroscopy. Chloride (Cl^-^) was determined by titrimetric analysis of precipitation (argentometric method). These methods were conducted in accordance with the Standard Methods for the Examination of Water and Wastewater [29].

### DNA extraction and metagenomic sequencing

Total DNA from fresh water was extracted using the PowerWater^®^ DNA Isolation kit (Mo Bio Inc. Carlsbad CA, USA) according to the manufacturer′s protocol, from approximately 300 mL of fresh water filtered onto a 0.22 μm membrane. Total DNA from soil was extracted from 50 mg of soil using a PowerLyzer^®^ PowerSoil^®^ DNA Isolation kit (Mo Bio Inc. Carlsbad CA, USA) according to the manufacturer′s protocol.

DNA extracts were checked for quality by agarose gel electrophoresis and with a 2100 Bioanalyzer (Agilent Technologies) and then quantified by fluorescence spectroscopy (Qubit). DNA samples were sequenced using the Ion Proton platform with chip PI and Ion PI Template OT2 200 v3 (Life Technologies) and Ion PI sequencing 200 v3 (Life Technologies) according to manufacturer’s protocol.

### Sequence analysis

Raw sequence reads were quality filtered and trimmed using the software PRINSEQ [30]. The sequence reads were trimmed with an average quality threshold of 20 or greater and checked using FastQC [31]. The quality filtered reads were submitted to annotation on MG-RAST metagenomics analysis server version 3.6 [32].

Microbial composition analysis was performed using the MG-RAST best hit classification tool, where reads were compared to the SSU-SILVA (non-redundant) database [33] using a maximum e-value of 1e-5, a minimum identity of 80%, and a minimum alignment length of 60, measured in bp, to generate taxonomic profiles.

Functional classification was performed using the MG-RAST hierarchical classification tool based on KEGG Orthology (KO) [34] and SEED Subsystems [35]. The data was compared to each database using a maximum e-value of 1e-5, a minimum identity of 80%, and a minimum alignment length of 20, measured in amino acids, to generate functional profiles.

To identify the potential for nitrogen metabolism in each sample, genes related to this biogeochemical cycle were selected within KO according to the threshold described above.

### Statistical analysis

Differential abundance of taxonomic groups and functional genes in soil and fresh water environments were determined using the R package ‘mvbund’ [36], after rarefying raw counts obtained from MG-RAST using the function *rrarefy* within the R package ‘vegan’ [37]. Negative binomial generalized linear models (nb GLMs) were created for each variable separately (given a strong mean-variance relationship) with environment as an explanatory factor (2 levels–Soil and Water). Likelihood ratio tests (LRTs) were used to test for significance of environment for each variable, and for multivariate hypothesis testing, the sum LRTs (sum of LR) from individual GLMs was used as a multivariate test of community differences [36]. *P values* were calculated using 999 bootstraps of residuals (resampling rows of the data to account for correlation between variables). Significantly differential variables were ordered by relative abundance to identify the features that contribute strongly to the overall difference between soil and fresh water samples, and visualized using barplots and heatmaps.

For an exploratory analysis of soil microbial communities, Bray-Curtis similarity distances were calculated between the soil samples studied here and 22 other soil samples from publicly available data retrieved from MG-RAST (S1 Table) using untransformed sequence counts. Result of similarity distances were visualized using non-metric multidimensional scaling (nMDS). The MG-RAST data were selected based on sequence type (shotgun metagenome) and feature (agricultural soil, cropland soil).

## Results and discussion

### Physical and chemical analysis of soil and fresh water

Analysis of the soil revealed it to be slightly acidic (pH = 5.57 ± 0.09), with high phosphorus (486.67 ± 45.39 mg/dm^3^), calcium (82.67 ± 7.86 mmol_c_/dm^3^) and iron (59 ± 3.00 mmol_c_/dm^3^) concentrations (S2 Table). The fresh water had a neutral pH (6.92 ± 0.01) and showed low concentrations of nitrogen (NH^+^_4_, NO^-^_3_ and NO^-^_2_ ≤ 1.02 mg L^-1^) and phosphorus (DRP and TP ≤ 0.16 mg L^-1^, S2 Table). In addition, there were low levels of chemical oxygen demand (4.33 ± 0.33 mg L^-1^), total dissolved solids (TDS, 27.67 ± 3.18 g L^-1^) and turbidity (136.67 ± 7.31 NTU) observed, showing that the fresh water had overall low nutrient concentrations (S3 Table). These fresh water conditions are not conducive to cyanobacterial and algal blooms, also suggesting that microorganisms are probably adapted to low nutrient conditions and store a substantial amount of products.

### Sequence processing, quality filtering, and annotation

High molecular weight genomic DNA was extracted from each sample and sequenced using the Ion Proton platform (Life Technologies). Each sample yielded over 2.316 x 10^7^ sequence reads (Table 1). After quality filtering, the minimum number of sequence reads per sample was 1.943 x 10^7^. The reads of the soil metagenome exhibited an average GC content higher than that of fresh water (Table 1). One intrinsic fact for this result can be correlated to the already known high complexity of the soil microorganisms. However, as stated by Foerstner et al [38] it is difficult to attribute the distribution of GC content just as a simple, unbiased mix of all prokaryotes known at the moment of analyses, because the environment has a considerable impact on GC content of samples.

**Table 1:** DNA sequence read metrics of the six metagenomic samples from soil from vegetable crop (SVG) and fresh water used for irrigation (FW) based on MG-RAST annotation.

Metagenome		Sequences count	Sequences count post quality control	Average GC content	Reads average length
Soil	SVG1	31,764,931	28,588,669	63 ± 8%	93 bp
	SVG2	44,539,294	42,109,184	63 ± 8%	93 bp
	SVG3	23,164,262	19,428,522	62 ± 9%	93 bp
Fresh water	FW1	32,542,115	26,668,719	46±12%	108 bp
	FW2	30,563,031	28,289,666	49±11%	109 bp
	FW3	48,438,622	41,940,721	49±12%	120 bp

### Taxonomic comparisons between environments

The analysis of the taxonomic community showed that the environments were dominated by Bacteria (97.6% in soil and 95.2% in fresh water). The remaining sequences matched with the Archaea (1% in soil and 0.2% in fresh water) and Eukaryota (0.9% in soil and 1.9% in fresh water) or were unassigned (0.5% in soil and 2.7% in fresh water).

**Fig 2:**
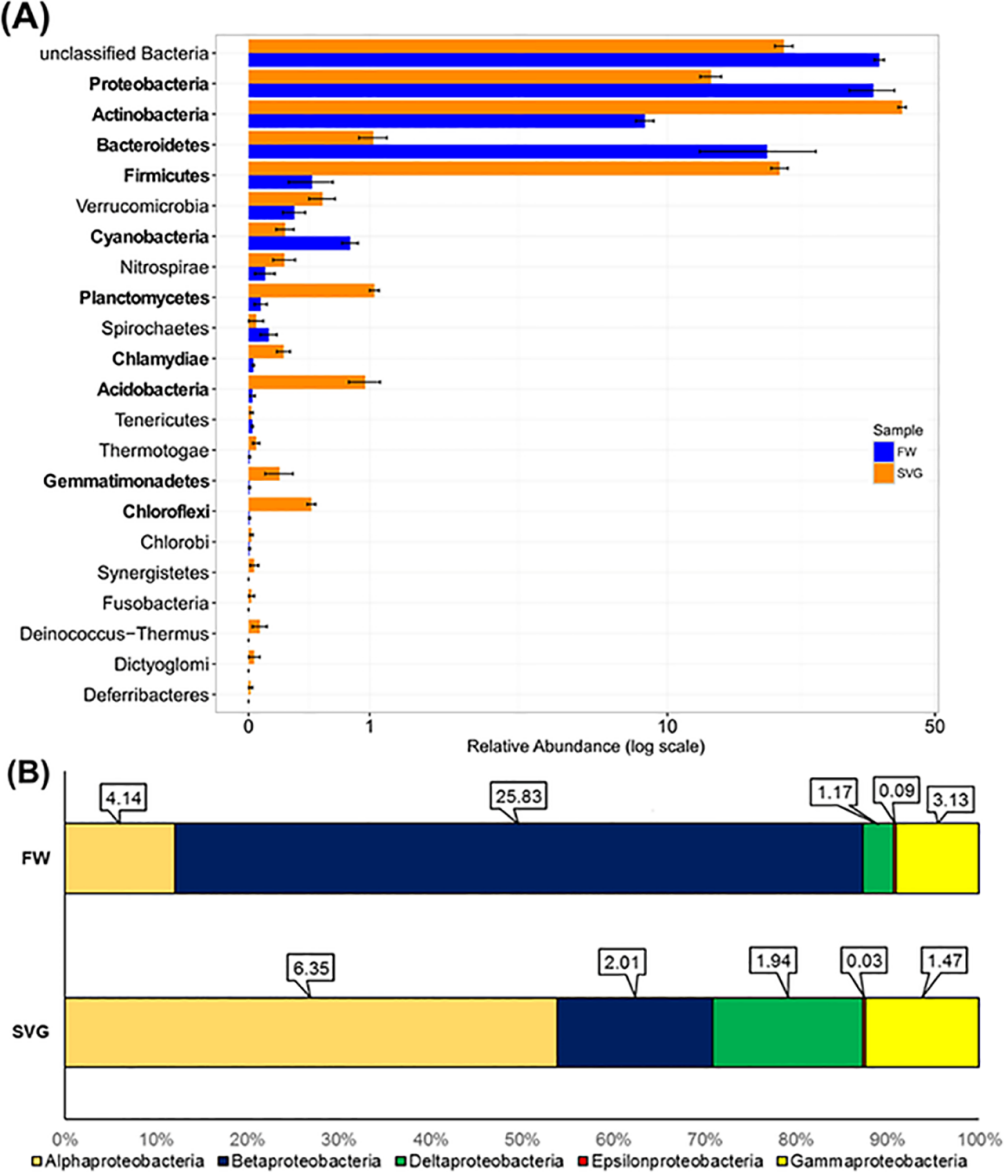
Relative abundance of bacterial taxa within microbial communities sampled from SVG and FW used for irrigation. (A) bar plots with standard error of the taxonomic distribution of the bacterial phyla. (B) relative abundance of Proteobacteria classes with the percentage values represented above the bars. The phyla names highlighted in bold indicate that they showed a significant difference between the two environments (p-value < 0.05).

The bacterial composition of samples from SVG and FW was further investigated using the Silva SSU database. A total of 22 phyla within the Bacterial domain were detected among the environments, however, a large proportion of the sequences were unclassified at the phylum level (approximately 40% of sequences within the fresh water samples and 20% for soil samples, Fig 2A). The relative abundance of the phyla Proteobacteria, Cyanobacteria and Bacteroidetes was significantly higher in FW compared to SVG (Fig 2A, see also S4 Table). The phyla Actinobacteria, Firmicutes, Planctomycetes, Gemmatimonadetes, Chloroflexi, and Acidobacteria were greater in relative abundance in SVG compared to FW (Fig 2A, see also S4 Table). Within the Proteobacteria (Fig 2B), Betaproteobacteria were relatively more abundant in FW sample, while SVG had more Alphaproteobacteria.

A total of 680 different bacterial genera were detected, of which 308 were present in both SVG and FW samples. Among all the genera found, 66 showed statistical differences between the two environments (S5 Table), of which 46 were within the shared genera found for SVG and FW samples. Despite some expected differences between the two samples from distinct environments, the results showed a high rate of shared bacterial genera among the samples analysed. These results are consistent with Ruiz-González [39] findings that support the idea that freshwater networks has a directional spatial structure driven by a common terrestrial origin, which results in local aquatic communities numerically dominated by terrestrially derived taxa.

**Fig 3:**
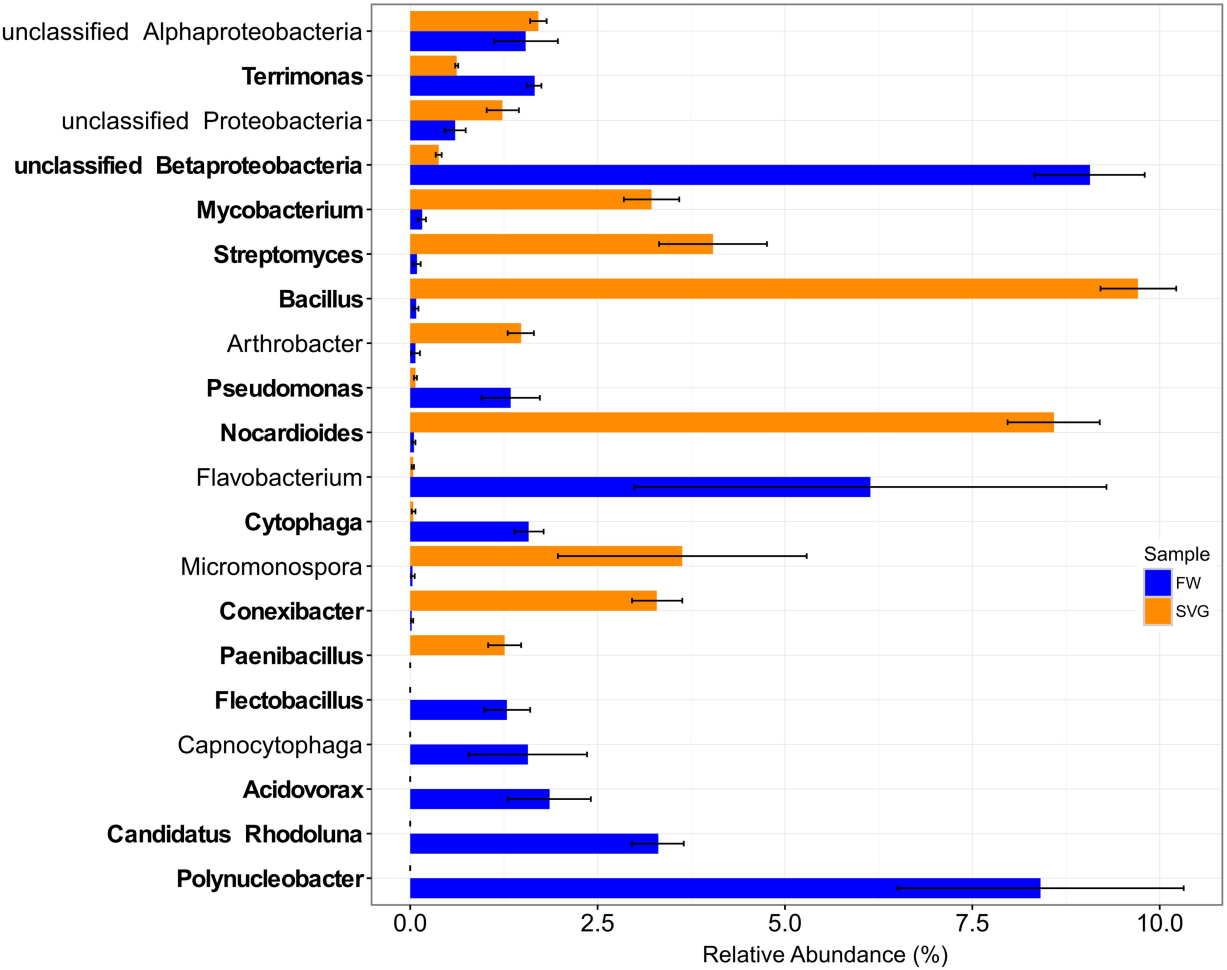
Relative abundance of bacterial genera within microbial communities sampled from SVG and FW. The figure displays a barplot with standard error of the taxonomic distribution of the twenty most abundant bacterial genera, with names highlighted in bold indicating genera that showed a significant difference between the two environments (p-value < 0.05).

The most abundant genera were *Polynucleobacter* (~ 8% relative abundance) in FW and *Bacillus* (~10%) in SVG. Comparison of the relative abundances between the environments showed that the genera *Mycobacterium*, *Streptomyces*, *Bacillus*, *Nocardioides*, *Conexibacter*, and *Paenibacillus* were in greater abundance in SVG compared to FW (Fig 3, see also S5 Table), while the *Terrimonas*, *Pseudomonas*, *Cytophaga*, *Flectobacillus*, *Acidovorax*, *Candidatus Rhodoluna*, *Polynucleobacter* and unclassified genera derived from Betaproteobacteria were in greater abundance in FW compared to SVG (Fig 3, see also S5 Table).

These results are consistent with previous studies that noted Betaproteobacteria as often numerically dominant in freshwater [40,41]. Some genera within this class, such as *Polynucleobacter* and *Limnohabitans*, are widely distributed in freshwater habitats and play an important ecological role in the food chain [42–44]. The genus *Polynucleobacter* is also frequently found in freshwater habitats and is one of the best-studied freshwater bacterium [40,41,43,44]. These oligotrophic ultramicrobacteria are specialized in utilising photo-oxidation products of humic substances, and some strains use light as an energy source [45,46]. In addition, another important characteristic of this bacterial group is that it is not associated with short-living algal blooms [45,46], and can thus be considered a good indicator for water quality. This is consistent with the fresh water sampled here being clear and transparent, without any visible cyanobacterial or algal bloom. However, cyanobacteria were found in both FW and in SVG samples, but the twenty cyanobacterial genera observed represented only around 0.38% of the relative abundance. The other representative genera found only for FW, such as *Candidatus Rhodoluna* and *Flectobacillus* are known to inhabit freshwater ecosystems, but little is known about their ecology [41,47].

Bacteria belonging to the phyla Actinobacteria and Firmicutes were abundant in SVG samples. Actinobacteria are often abundant in soils under organic amendments [13,48] and its members are often associated with degradation of recalcitrant carbon compounds. The abundance of the genus *Bacillus* in SVG samples was not surprising as they are common microorganisms found in several agricultural soils and are important for biomass degradation and phosphorus solubilization [49,50]. As the second most abundant and statistically significant genus observed in this study, *Nocardioides* are characterized as a well-known group for xenobiotic compost degradation [51,52] and lignocellulose decomposer [53]. Also, its abundance was observed in a soil area treated with biochar-mineral complexes and compost based on poultry manure [9]. Moreover, this bacterial genus was one of the most abundant in soil where tobacco grew well without bacterial wilt (characterized as healthy) indicating a significant difference (p-value < 0.05) between healthy and bacterial wilt infected soil [54]. Also, among the most abundant bacterial genera, *Paenibacillus* was found only in SVG samples. This group is known as Plant-Growth-Promoting Rhizobacteria (PGPR) which produce phytohormones (indole-3-acetic acid), solubilize phosphate and some strains also have been used to control plant pathogenic microorganisms [55–58]. Thus, the presence of *Paenibacillus* in SVG samples highlight its importance in agricultural soils to help in soil fertility, ecology and environmental biotechnological potential as demonstrated by several studies that isolated different strains of this bacteria from a variety of soil [57,59,60].

**Fig 4:**
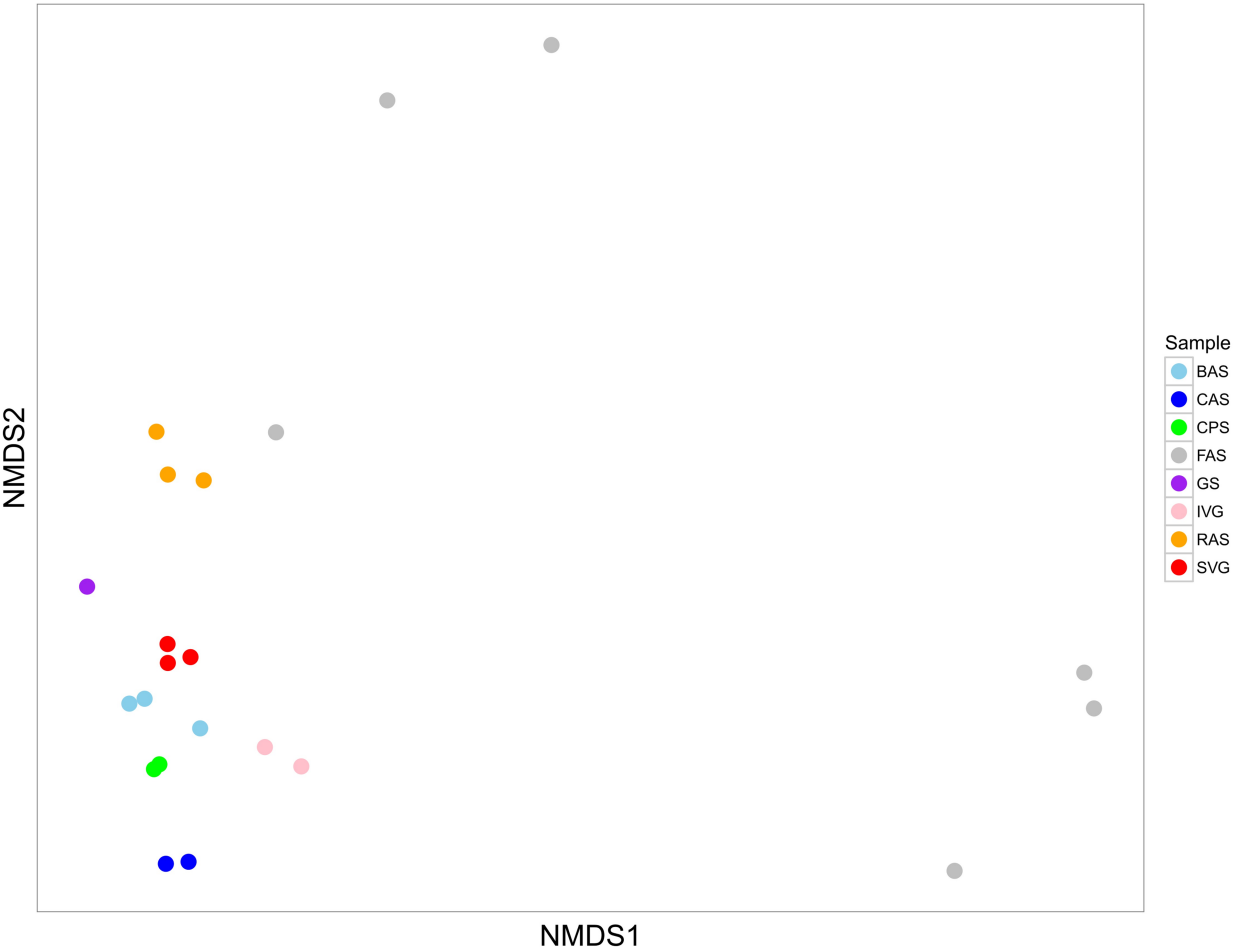
Non-metric multidimensional scaling (NMDS) based on genera taxonomic distribution of different agricultural soil metagenomes publicly available on MG-RAST. **Samples were compared using the Bray Curtis similarity distance. Stress 0.0833**. The agricultural soil points shown in this figure are related to samples from different countries: Brazilian bulk agricultural soil (BAS), Canada agricultural soil (CAS), China agricultural soil (CPS), France agricultural soil (FAS), Germany soil (GS), Israel vegetable garden soil (IVG), rhizosphere soil (RAS), and soil from vegetable crops (SVG).

Furthermore, based on an exploratory analysis our taxonomic results at the genus level showed the SVG samples were closely related to other agricultural soil samples (Fig 4). This suggests that even receiving organic compost made from vegetable and animal residues (including carcass), the soil samples analysed here had a bacterial community similar to those found in soil from other agricultural areas.

**Fig 5:**
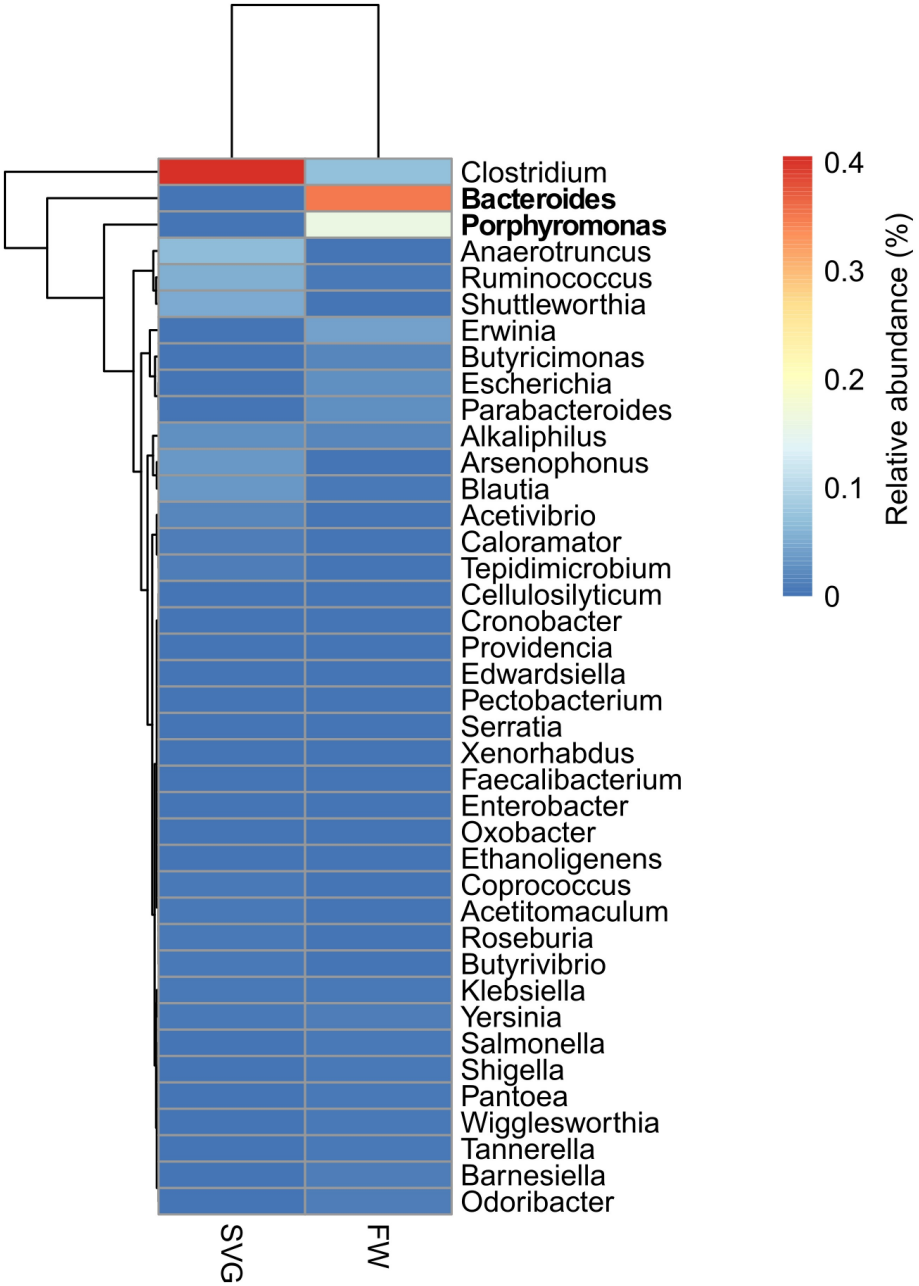
Mean relative abundances of the bacterial genera used as faecal indictors within the metagenomes from SVG and FW. The figure displays a heatmap of the 40 faecal bacterial communities found, with names highlighted in bold indicating genera that showed a significant difference between the two environments at p-value < 0.05.

In agricultural areas, irrigation water is a key factor for crop production. Surface fresh water from shallow streams and small rivers are commonly used for irrigation due to their proximity and ease with which they can be used in farm fields. However, these systems are frequently exposed to contamination by faecal, sewage and pesticides pollution [61–63]. There are many studies confirming irrigation water as a source of pathogens on fresh fruits and vegetables produce that evidence the necessity and importance of monitoring irrigation water due to the persistent risk to consumers health [61,62,64,65]. Because of that we also investigated the abundance of bacterial taxa that might be associated with faecal contamination in FW and SVG, such as genera from families *Bacteroidaceae*, *Porphyromonadaceae*, *Clostridiaceae*, *Lachnospiraceae*, *Ruminococcaceae* and *Enterobacteriaceae*, which have been proposed as a faecal signature [20]. These groups were retrieved directly from the total bacterial genera rarefied data previous calculated and transformed into relative abundance. The bacterial pathogens affiliated with faecal contamination were found in low abundance, less than 0.4% of the total bacterial genera identified (Fig 5). *Bacteroides* and *Porphyromonas* were found only in FW, suggesting that these bacterial genera did not establish themselves in the soil, despite their input through irrigation. Similarly, the genus *Escherichia* was found in very low abundance in FW and is absent in SVG. Detection of pathogenic *Escherichia coli* is commonly used as an indicator of faecal contamination in waterways [66,67]. Based on the obtained results, we suggest that the irrigation fresh water analysed here has a minimal risk for introducing faecal pathogens and can thus be considered appropriate to irrigate vegetables.

### Functional potential of environments

**Fig 6:**
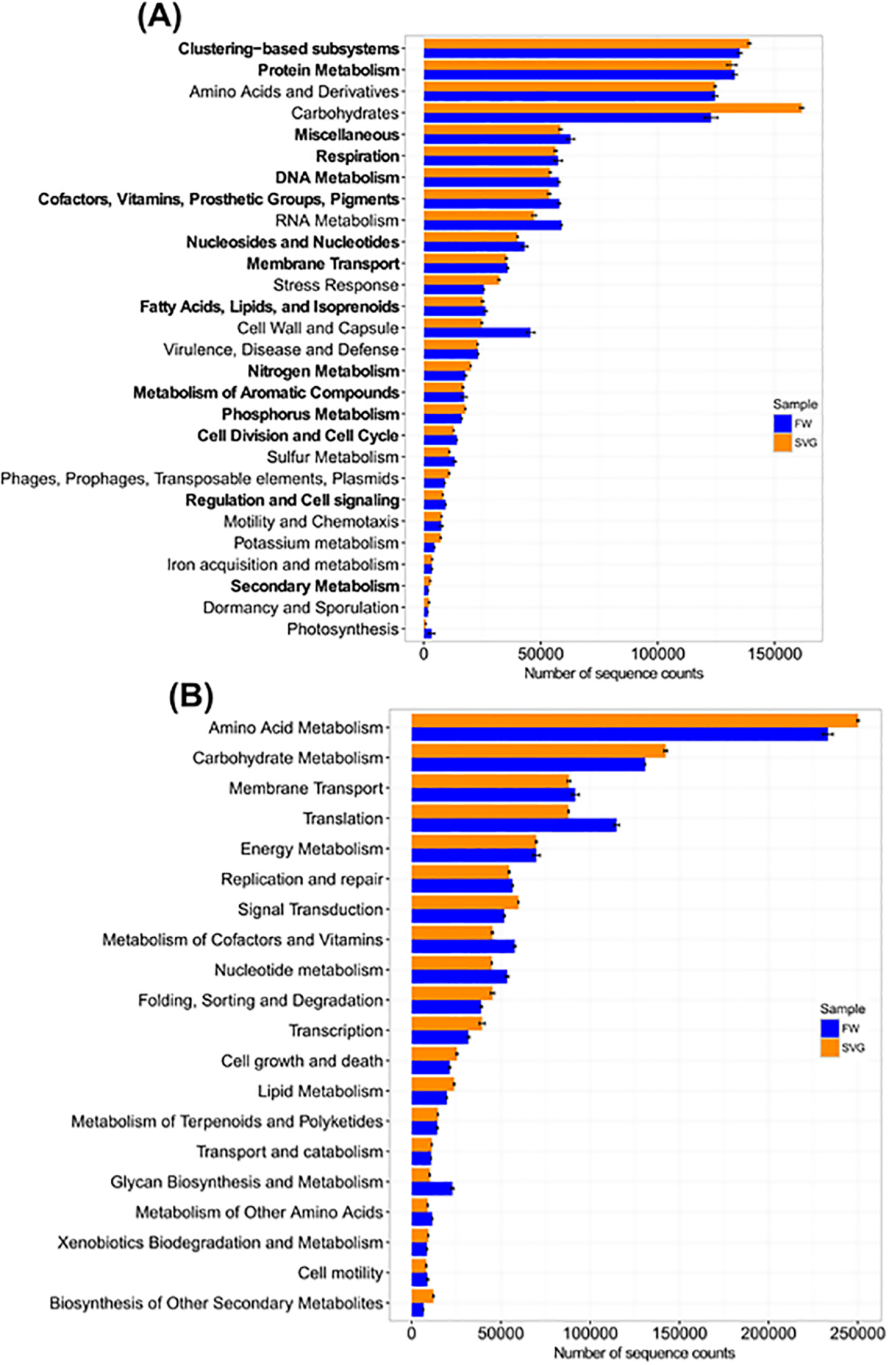
Comparison of the functional profiles for the microbial metagenomes of the SVG and FW. The figure displays the number of normalized sequence counts found for each metagenome for (A) SEED Subsystems and (B) KEGG Orthology annotation. Categories highlighted in bold indicate a significant difference between the two environments (p-value < 0.05).

The metagenomic sequencing provided insights into the lifestyle and metabolic potential of organisms inhabiting SVG and FW. From the sequences in each metagenome, predicted proteins were annotated using the SEED Subsystems (Fig 6A) and KEGG Orthology (Fig 6B).

At the level of SEED Subsystems, except for the functional categories associated with carbohydrate metabolism and cell wall and capsule formation, only slight variations were observed between the different environmental samples. Carbohydrate metabolism, which includes functional genes related to biomass degradation, dominated the SVG samples. This result is consistent with the taxonomic analysis, in which we found great abundance of Actinobacteria and Firmicutes, bacterial groups directly related to biomass degradation.

Looking specifically at the nitrogen and phosphorus metabolism, due to their importance for agriculture, we observed a higher number of sequence counts in SVG for both subsystems. This occurred probably because of the greater concentration of nutrients present in soil (S2 Table).

A more detailed evaluation of the sequences related to phosphorus metabolism revealed a predominance of genes linked to phosphate metabolism, suggesting the direct solubilization of phosphorus and, therefore, immediate availability to the crops [68,69]. This can also explain the high abundance of *Bacillus*, considering several strains of these bacteria are known to be important phosphate-solubilizing microorganisms [50,69,70].

Among all the subsystems that showed p-value < 0.05, one interesting finding was regarding the annotation of sequences related to metabolism of aromatic compounds, which revealed a predominance of genes linked to the benzoate transport and degradation cluster. It is known that the aromatic degradation pathways are an important source of metabolic exchange factors for microorganisms that can use different aromatic compounds as sole carbon and energy sources [71]. Benzoate is a chemical compound present in some pesticides which is persistent in the environment [72,73]. This result could suggest a possible runoff from soil into the fresh water due to the influence of agricultural practices, as also stated by Lopes et al [74].

The KEGG Orthology (KO) data indicated high similarity between the functional categories analysed for SVG and FW, but some differences in translation, glycan biosynthesis and metabolism categories (Fig 6B).

### Comparisons of the nitrogen metabolism of the environments

**Fig 7:**
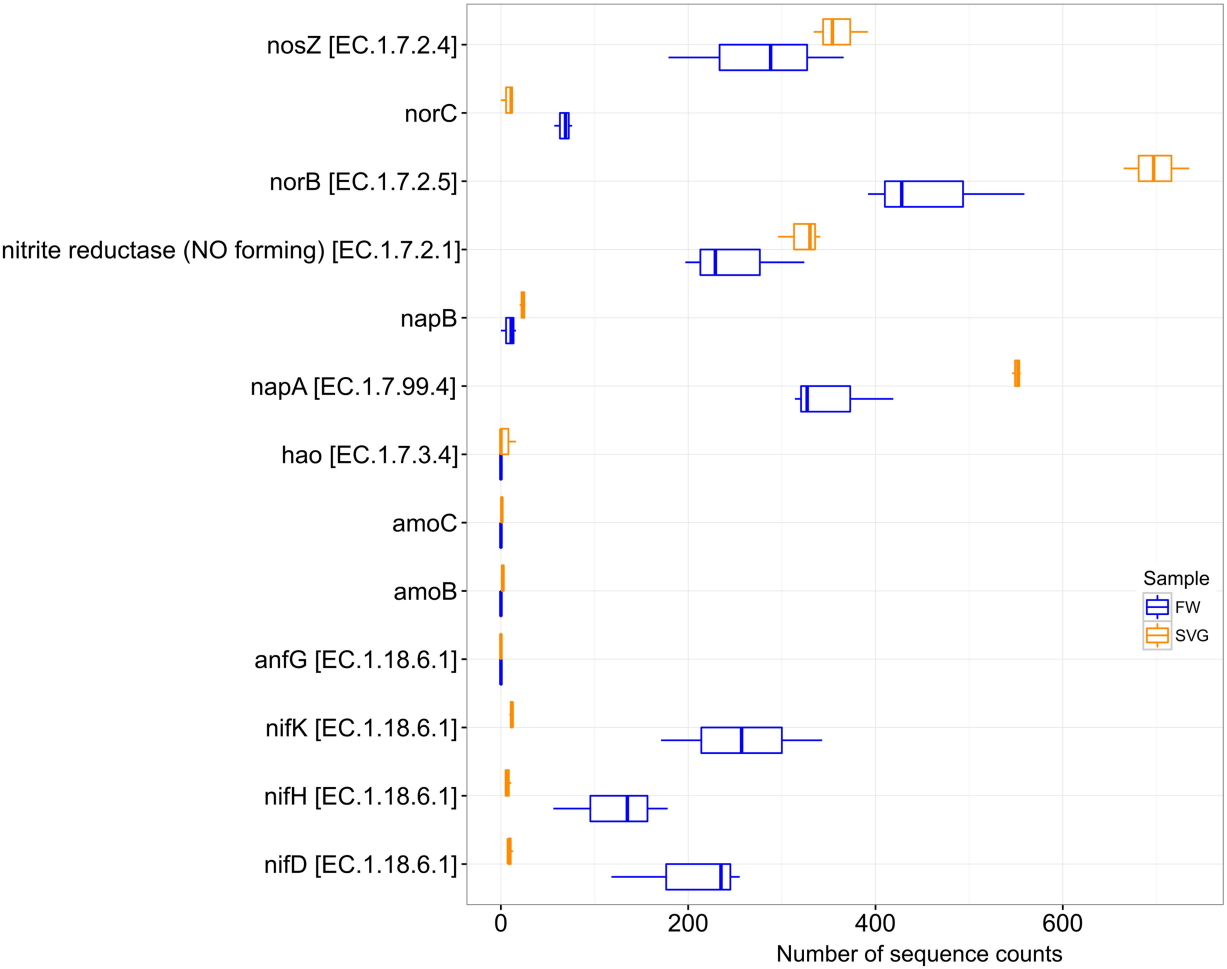
Distribution of nitrogen metabolism genes among the samples from SVG and FW. The figure displays a boxplot with number of sequence counts of each gene present in the samples analyzed according the KO annotation.

The functional profiles of SVG and FW samples were further analysed for genes predicted to be linked to nitrogen metabolism based on KO assignments (Fig 7). SVG samples had a high abundance of genes related to denitrification processes, such *norB* (nitric oxide reductase subunit B), *napA* (periplasmic nitrate reductase), *nitrite reductase (NO-forming)* and *nosZ* (nitrous-oxide reductase). FW samples showed a higher abundance of genes related to N-fixation process, such *nifHDK* (nitrogen fixation protein cluster) compared to the SVG samples. The genes *amo* (ammonia monooxygenase) and *hao* (hydroxylamine oxidase) for the aerobic and anaerobic nitrification pathway were only found in SVG samples, but in low relative abundance (S6 Table).

From the distribution of genes related to the nitrogen cycle, a high abundance of *norB* (large subunit of nitric oxide reductase) gene was observed. This gene catalyses the reduction of NO to N_2_O. It can be found in a variety of microorganisms including denitrifying and non-denitrifying bacteria [75]. In contrast, for FW there was a low concentration of organic matter and a high concentration of dissolved oxygen, but also a high abundance of denitrifying genes. This can suggest the presence of microorganisms able to perform aerobic denitrification, since a substantial rate of redox potential (212.00 ± 27.51 mV) was observed. Also, from the abundance of *nifDKH* in FW samples, it seems that the N-fixation is playing a crucial role in freshwater. This seems to occur due to the nitrogen limitation, as indicated by the low concentrations of nitrogen found in the freshwater, and thus suggesting incomplete nitrogen pathways in both SVG and FW.

### Detection of potential virulence genes

Due to the importance of the aforementioned monitoring of irrigation water, the functional categories associated with virulence and antibiotic resistance genes for FW and SVG were determined by the classification of predicted functional genes based on SEED subsystems. The importance to screen these potential virulence features by NGS based methods rely on the investigation of potential risk agents for humans and other animals [21,23], as concluded by Jongman and Korsten [63] surface water may be a possible preharvest source of contamination on leafy green vegetables that may comprise a health risk to consumers.

**Fig 8:**
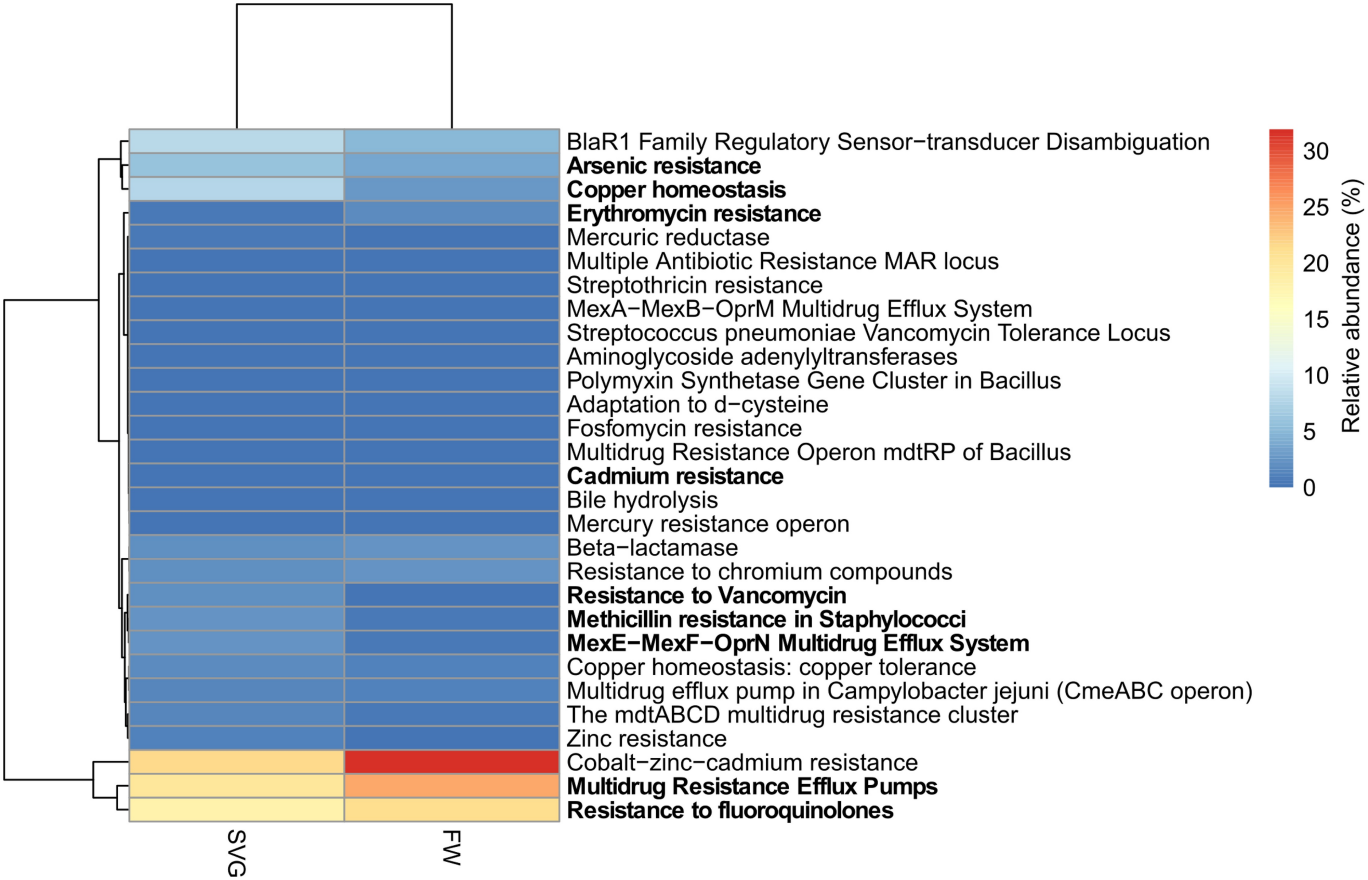
Resistance to antibiotics and toxic compound related functions within the metagenomes from SVG and FW. The figure displays a heatmap with relative abundance of the features found and highlighted in bold when a significant difference was found between two environments at a p-value < 0.01.

Table 2 presents the results obtained from the categories associated with virulence, disease and defense within SEED subsystems. The functional category for resistance to antibiotics and toxic compounds was the most abundant in both samples analysed. The abundance of genes related to metal resistance, such as cobalt, zinc and cadmium resistance, was also similar for SVG and FW (Fig 8, see also S7 Table). Also, there was a substantial abundance of functional categories associated with resistance to fluoroquinolones and multidrug resistance efflux pumps for SVG and FW (Fig 8, see also S7 Table). As concluded by Durso et al [76], antibiotic resistance genes are common in agricultural and non-agricultural habitats as their presence is often related to functionally important mechanisms in many habitats, and may not be uniquely interpreted as a harmful finding for this ecosystem. However, an intriguing observation can be made based on the results herein, it seems that the management followed in this particular agricultural area associated with the Zoo is increasing the resistance of microorganisms in both the freshwater stream and soil to fluoroquinoles, multiple drugs and Co-Zn-Cd.

**Table 2:** Relative abundance of functional categories associated with virulence, disease and defense found in soil from vegetable crop (SVG) and fresh water used for irrigation (FW) according to SEED Subsystems annotation.

Function category	SVG	FW
Adhesion*	0.01322	0.00808
Bacteriocins, ribosomally synthesized antibacterial peptides**	0.00589	0.00193
Detection**	0.02122	0.00385
Invasion and intracellular resistance*	0.00046	0.00110
Resistance to antibiotics and toxic compounds**	0.84820	0.89900
Toxins and superantigens**	0.00382	0.00113

**significant at p-value < 0.01

*significant at p-value < 0.05

Furthermore, an abundance of sequence reads related to pathogenicity island genes were detected in both samples (S8 Table). Pathogenicity islands are distinct genetic elements on the chromosomes, often found in a large number of bacterial pathogens, where they are acquired by horizontal gene transfer. Pathogenicity islands encode a diverse set of virulence and pathogenic factors, but they are normally absent from non-pathogenic strains of the same or closely related species [77,78]. It is known that bacterial diversity and complexity is greater in soil than in freshwater ecosystems [39] and this can most likely provide a suitable situation for gene transfer [78].

Enteropathogenic *E*. *coli* (EPEC) and enterohemorrhagic *E*. *coli* (EHEC) can cause severe diarrheal problems as well as potentially fatal human and veterinary diseases [66]. As the genus *Escherichia* was present in low abundance in FW, we searched for genes related to enteropathogenic *Escherichia coli* infection EPEC/EHEC. However, we did not detect any genes related to this pathogenic feature for any of the samples, indicating that the *E*. *coli* in the irrigation system might not be pathogenic.

To our knowledge, this is one of the few studies describing the microbiome of soil and fresh water under fertilization treatment with an organic compost made of vegetable and animal residues, such as carcass. Although the findings of this study cannot explain how the organic compost used in the area influenced the current microbial aspects in soil or fresh water, it provided a good understanding of the microbial agricultural ecology in the area. From the perspective of soil fertilization with animal-related material in organic amendments, there is concern about the possible introduction of potential pathogenic agents to human and animals in soil [79,80], the results herein showed the soil had common bacterial groups already found in other agricultural soil areas under different organic amendment treatments. Furthermore, the results obtained showed that the quality of freshwater used for crop irrigation seems to be adequate for such use, since we did not detect toxigenic related genes, faecal contaminators, bloom-forming cyanobacteria or an overload of nitrogen and phosphorus nutrients.

## Conclusion

This study provided the basis for a better understanding of the microbial community structure of an area under strong agricultural activity with a regular irrigation process and fertilization with organic compost made of animal carcasses. The soil from vegetable crops and the fresh water used for irrigation showed common bacterial groups also seen in other agricultural systems. Our microbial analysis of the freshwater showed that the water stream analysed here was suitable for irrigation purposes. In addition, the use of a metagenomic approach to check the freshwater quality proved to be a suitable method to investigate the presence, diversity and potential role of pathogenic and virulent microorganisms in agricultural ecosystems. However, this methodology still can’t replace PCR-based methods targeting specific virulence genes to confirm the expression of such genes in genetic material extracted from total microorganisms of environmental samples, perhaps both techniques could be used in parallel.

## Supporting information

S1 Table (DOCX)

S2 Table (DOCX)

S3 Table (DOCX)

S4 Table (DOCX)

S5 Table (DOCX)

S6 Table (DOCX)

S7 Table (DOCX)

S8 Table (DOCX)
