# Supplementary material for: Metagenomic analysis of soil and freshwater from zoo agricultural area with organic fertilization
Source: PLoS One. 2017 Dec 21;12(12):e0190178. doi: 10.1371/journal.pone.0190178 (PMC5739480; doi:10.1371/journal.pone.0190178)
Supplement: S1 Table — (DOCX) [file pone.0190178.s001.docx]

Table S1. MG-RAST reference data used in the NMDS analysis.

| MG-RAST ID | Project ID | Material | Feature | Country | Legend |
| --- | --- | --- | --- | --- | --- |
| mgm4497370.3 | mgp1519 | bulk soil | agricultural soil | Brazil | BAS |
| mgm4497371.3 | mgp1519 | bulk soil | agricultural soil | Brazil | BAS |
| mgm4497387.3 | mgp1519 | bulk soil | agricultural soil | Brazil | BAS |
| mgm4497379.3 | mgp1519 | bulk soil | forest | Brazil | BF |
| mgm4497380.3 | mgp1519 | bulk soil | forest | Brazil | BF |
| mgm4497381.3 | mgp1519 | bulk soil | forest | Brazil | BF |
| mgm4615272.3 | mgp12156 | rhizosphere | agricultural soil | Brazil | RAS |
| mgm4615241.3 | mgp12156 | rhizosphere | agricultural soil | Brazil | RAS |
| mgm4615274.3 | mgp12156 | rhizosphere | agricultural soil | Brazil | RAS |
| mgm4482600.3 | mgp8744 | soil | agricultural soil | Canada | CAS |
| mgm4483819.3 | mgp8744 | soil | agricultural soil | Canada | CAS |
| mgm4620533.3 | mgp12555 | soil | agricultural soil | France | FAS |
| mgm4620529.3 | mgp12555 | soil | agricultural soil | France | FAS |
| mgm4620535.3 | mgp12555 | soil | agricultural soil | France | FAS |
| mgm4685220.3 | mgp16910 | soil | soil | Germany | GS |
| mgm4689226.3 | mgp17193 | soil | paddy field soil | China | CPS |
| mgm4696917.3 | mgp17193 | soil | paddy field soil | China | CPS |
| mgm4697958.3 | mgp18015 | soil | agriculre soil | France | FAS |
| mgm4705034.3 | mgp18015 | soil | agriculre soil | France | FAS |
| mgm4705038.3 | mgp18015 | soil | agriculre soil | France | FAS |
| mgm4729577.3 | mgp21199 | clay soil | vegetable garden soil | Israel | IVG |
| mgm4729578.3 | mgp21199 | clay soil | vegetable garden soil | Israel | IVG |
