# Supplementary material for: Metagenomic analysis of soil and freshwater from zoo agricultural area with organic fertilization
Source: PLoS One. 2017 Dec 21;12(12):e0190178. doi: 10.1371/journal.pone.0190178 (PMC5739480; doi:10.1371/journal.pone.0190178)
Supplement: S2 Table — (DOCX) [file pone.0190178.s002.docx]

S2 Table. Mean ± standard error (n =3) chemical properties of the soil from vegetable crop (SVG).

| Variable | Measurement |
| --- | --- |
| pH (CaCl_2_) | 5.57 ± 0.09 |
| Organic Matter (g/dm³) | 40.33 ± 0.88 |
| P (mg/dm³) | 486.67 ± 45.39 |
| K (mmol_c_/dm³) | 4.57 ± 0.07 |
| Ca (mmol_c_/dm³) | 82.67 ± 7.86 |
| Mg (mmol_c_/dm³) | 19.67 ± 1.20 |
| B (mmol_c_/dm³) | 0.45 ± 0.02 |
| Cu (mmol_c_/dm³) | 0.43 ± 0.03 |
| Fe (mmol_c_/dm³) | 59 ± 3.00 |
| Mn (mmol_c_/dm³) | 1.70 ± 0.12 |
| Zn (mmol_c_/dm³) | 7.23 ± 0.23 |
