# Supplementary material for: Metagenomic analysis of soil and freshwater from zoo agricultural area with organic fertilization
Source: PLoS One. 2017 Dec 21;12(12):e0190178. doi: 10.1371/journal.pone.0190178 (PMC5739480; doi:10.1371/journal.pone.0190178)
Supplement: S3 Table — (DOCX) [file pone.0190178.s003.docx]

S3 Table. Mean ± standard error (n =3) chemical and physical properties of the freshwater used for irrigation (FW).

| Measurement | FW |
| --- | --- |
| NO^-^_2_ (mg L^-1^) | 0.06 ± 0.00 |
| NO^-^_3_ (mg L^-1^) | 1.02 ± 0.01 |
| NH^+^_4_ (mg L^-1^) | 0.04 ± 0.00 |
| TP (mg L^-1^) | 0.16 ± 0.00 |
| DRP (mg L^-1^) | 0.02 ± 0.00 |
| COD (mg L^-1^) | 4.33 ± 0.33 |
| Temperature (°C) | 22.26 ± 0.77 |
| pH | 6.92 ± 0.01 |
| Dissolved oxygen (mg L^-1^) | 7.37 ± 0.66 |
| EC (µS cm^-1^) | 41.67 ± 5.61 |
| Turbidity (NTU) | 136.67 ± 7.31 |
| ORP (mV) | 212.00 ± 27.51 |
| TSD (g L^-1^) | 27.67 ± 3.18 |

TP: Total phosphorus; DRP: Dissolved reactive phosphorus; COD: Chemical Oxygen Demand; EC: electric conductivity; ORP: Redox potential; TDS: Total solid dissolved.
