# Supplementary material for: Metagenomic analysis of soil and freshwater from zoo agricultural area with organic fertilization
Source: PLoS One. 2017 Dec 21;12(12):e0190178. doi: 10.1371/journal.pone.0190178 (PMC5739480; doi:10.1371/journal.pone.0190178)
Supplement: S4 Table — (DOCX) [file pone.0190178.s004.docx]

S4 Table. Taxonomic counts of phylum in soil from vegetable crop (SVG1, SVG2 and SVG3) and freshwater used for irrigation (FW1, FW2 and FW3) bacterial metagenomes. The values are the sequence normalized counts for each sample. P values were calculated using 999 bootstraps of residuals (resampling rows of the data to account for correlation between variables). Data in table is sorted in ascending order of the p-value.

| Phylum | SVG1 | SVG2 | SVG3 | FW1 | FW2 | FW3 | p-value |
| --- | --- | --- | --- | --- | --- | --- | --- |
| Acidobacteria | 68 | 36 | 67 | 1 | 0 | 3 | 0.001 |
| Actinobacteria | 2526 | 2363 | 2539 | 570 | 466 | 527 | 0.001 |
| Bacteroidetes | 50 | 82 | 56 | 384 | 1644 | 1304 | 0.001 |
| Chlamydiae | 19 | 10 | 11 | 2 | 2 | 1 | 0.001 |
| Chloroflexi | 23 | 30 | 25 | 1 | 0 | 0 | 0.001 |
| Firmicutes | 1312 | 1106 | 1172 | 33 | 5 | 41 | 0.001 |
| Planctomycetes | 70 | 59 | 61 | 9 | 2 | 2 | 0.001 |
| Proteobacteria | 766 | 887 | 712 | 2643 | 1775 | 1852 | 0.001 |
| Cyanobacteria | 8 | 21 | 13 | 57 | 44 | 41 | 0.002 |
| Gemmatimonadetes | 7 | 23 | 5 | 1 | 0 | 0 | 0.032 |
| Deinococcus-Thermus | 0 | 3 | 9 | 0 | 0 | 0 | 0.296 |
| Thermotogae | 2 | 5 | 1 | 1 | 0 | 0 | 0.296 |
| Synergistetes | 5 | 0 | 1 | 0 | 0 | 0 | 0.43 |
| unclassified Bacteria | 1102 | 1325 | 1259 | 2259 | 2045 | 2186 | 0.53 |
| Verrucomicrobia | 18 | 39 | 38 | 24 | 8 | 22 | 0.63 |
| Deferribacteres | 2 | 0 | 0 | 0 | 0 | 0 | 0.719 |
| Dictyoglomi | 6 | 0 | 0 | 0 | 0 | 0 | 0.73 |
| Fusobacteria | 3 | 0 | 0 | 0 | 0 | 0 | 0.73 |
| Nitrospirae | 9 | 9 | 23 | 0 | 5 | 13 | 0.746 |
| Chlorobi | 2 | 1 | 0 | 1 | 0 | 0 | 0.777 |
| Spirochaetes | 0 | 0 | 8 | 13 | 2 | 7 | 0.777 |
| Tenericutes | 2 | 1 | 0 | 1 | 2 | 1 | 0.795 |
| Lentisphaerae | 0 | 0 | 0 | 0 | 0 | 0 | 1 |
