# Supplementary material for: Metagenomic analysis of soil and freshwater from zoo agricultural area with organic fertilization
Source: PLoS One. 2017 Dec 21;12(12):e0190178. doi: 10.1371/journal.pone.0190178 (PMC5739480; doi:10.1371/journal.pone.0190178)
Supplement: S7 Table — (DOCX) [file pone.0190178.s007.docx]

S7 Table. Counts of the genes related to resistance to antibiotics and toxic compounds from vegetable crop (SVG1, SVG2 and SVG3) and freshwater used for irrigation (FW1, FW2 and FW3) metagenomes accordingly to SEED Subsystems annotation. The values are the sequence normalized counts for each sample. P values were calculated using 999 bootstraps of residuals (resampling rows of the data to account for correlation between variables). Data in table is sorted in ascending order of the p-value.

| Genes | SVG1 | SVG2 | SVG3 | FW1 | FW2 | FW3 | p-value |
| --- | --- | --- | --- | --- | --- | --- | --- |
| Copper homeostasis | 1577 | 1501 | 1489 | 497 | 574 | 532 | 0.001 |
| Methicillin resistance in Staphylococci | 479 | 458 | 475 | 100 | 103 | 67 | 0.001 |
| Resistance to fluoroquinolones | 3410 | 3445 | 3786 | 3933 | 4447 | 4091 | 0.001 |
| Resistance to Vancomycin | 436 | 441 | 407 | 6 | 2 | 4 | 0.001 |
| Cadmium resistance | 25 | 33 | 41 | 1 | 0 | 3 | 0.002 |
| Erythromycin resistance | 66 | 89 | 101 | 388 | 250 | 313 | 0.002 |
| MexE-MexF-OprN Multidrug Efflux System | 400 | 428 | 570 | 51 | 97 | 87 | 0.002 |
| Multidrug Resistance Efflux Pumps | 3834 | 4030 | 3716 | 4719 | 4882 | 4792 | 0.004 |
| Arsenic resistance | 1152 | 997 | 1143 | 654 | 635 | 697 | 0.006 |
| Adaptation to d-cysteine | 18 | 11 | 23 | 4 | 2 | 3 | 0.012 |
| Multidrug Resistance Operon mdtRP of Bacillus | 17 | 19 | 5 | 1 | 0 | 0 | 0.024 |
| Mercury resistance operon | 28 | 32 | 38 | 17 | 19 | 16 | 0.025 |
| Zinc resistance | 316 | 269 | 142 | 84 | 25 | 43 | 0.038 |
| Aminoglycoside adenylyltransferases | 1 | 3 | 4 | 0 | 0 | 0 | 0.045 |
| BlaR1 Family Regulatory Sensor-transducer Disambiguation | 1586 | 1611 | 1499 | 1063 | 866 | 983 | 0.116 |
| Copper homeostasis: copper tolerance | 302 | 292 | 356 | 255 | 237 | 203 | 0.249 |
| Fosfomycin resistance | 2 | 3 | 13 | 1 | 0 | 1 | 0.277 |
| The mdtABCD multidrug resistance cluster | 388 | 178 | 328 | 38 | 162 | 135 | 0.351 |
| Mercuric reductase | 75 | 66 | 111 | 14 | 52 | 2 | 0.575 |
| Beta-lactamase | 425 | 358 | 472 | 452 | 418 | 463 | 0.741 |
| Bile hydrolysis | 46 | 29 | 22 | 18 | 11 | 0 | 0.741 |
| Multidrug efflux pump in Campylobacter jejuni (CmeABC operon) | 408 | 307 | 250 | 285 | 232 | 252 | 0.741 |
| Multiple Antibiotic Resistance MAR locus | 0 | 0 | 1 | 5 | 0 | 1 | 0.741 |
| Streptothricin resistance | 0 | 2 | 0 | 0 | 0 | 0 | 0.741 |
| MexA-MexB-OprM Multidrug Efflux System | 0 | 0 | 0 | 0 | 1 | 0 | 0.792 |
| Streptococcus pneumoniae Vancomycin Tolerance Locus | 1 | 0 | 0 | 0 | 0 | 0 | 0.792 |
| Cobalt-zinc-cadmium resistance | 4063 | 4418 | 4082 | 6504 | 6019 | 6245 | 0.819 |
| Polymyxin Synthetase Gene Cluster in Bacillus | 11 | 0 | 0 | 5 | 1 | 0 | 0.819 |
| Resistance to chromium compounds | 404 | 450 | 396 | 375 | 435 | 537 | 0.819 |
