# Supplementary material for: Metagenomic analysis of soil and freshwater from zoo agricultural area with organic fertilization
Source: PLoS One. 2017 Dec 21;12(12):e0190178. doi: 10.1371/journal.pone.0190178 (PMC5739480; doi:10.1371/journal.pone.0190178)
Supplement: S8 Table — (DOCX) [file pone.0190178.s008.docx]

S8 Table. Counts of the genes for pathogenicity island found in the soil vegetable crop (SVG1, SVG2 and SVG3) and freshwater used for irrigation (FW1, FW2 and FW3) metagenomes accordingly to SEED Subsystems annotation. The values are the sequence normalized counts for each sample.

| Function | Gene | SVG1 | SVG2 | SVG3 | FW1 | FW2 | FW3 |
| --- | --- | --- | --- | --- | --- | --- | --- |
| Listeria Pathogenicity Island  LIPI-1 extended | Broad-substrate range phospholipase C (EC 3.1.4.3) | 0 | 1 | 1 | 0 | 0 | 0 |
|  | Phosphatidylinositol-specific phospholipase C (EC 4.6.1.13) | 2 | 0 | 1 | 0 | 1 | 0 |
|  | Thiol-activated cytolysin | 3 | 1 | 2 | 0 | 0 | 0 |
|  | Zinc metalloproteinase precursor (EC 3.4.24.29) | 1 | 2 | 1 | 0 | 0 | 1 |
|  | virulence cluster protein B VclB | 0 | 0 | 0 | 0 | 0 | 0 |
| Staphylococcal pathogenicity islands SaPI | GMP synthase [glutamine-hydrolyzing] (EC 6.3.5.2) | 1656 | 1482 | 1515 | 1631 | 2036 | 1933 |
|  | Heat shock protein 60 family chaperone GroEL | 3370 | 3569 | 3539 | 3115 | 2809 | 2901 |
|  | Hypothetical SAV0801 homolog in superantigen-encoding pathogenicity islands SaPI | 0 | 0 | 0 | 0 | 0 | 0 |
|  | Methionine ABC transporter substrate-binding protein | 29 | 15 | 22 | 8 | 11 | 6 |
|  | SSU ribosomal protein S18p | 215 | 185 | 170 | 229 | 243 | 229 |
|  | tmRNA-binding protein SmpB | 77 | 94 | 99 | 372 | 256 | 284 |
| Vibrio pathogenicity island | Cytoplasmic, CoA-independent, aldehyde dehydrogenase (EC 1.2.1.3) | 3 | 5 | 6 | 1 | 0 | 0 |
|  | Lipoprotein, ToxR-activated gene, TagA | 0 | 2 | 0 | 0 | 0 | 2 |
|  | TCP pilin signal peptidase, TcpA processing | 0 | 0 | 0 | 0 | 0 | 0 |
|  | Toxin co-regulated pilus biosynthesis protein I, chemoreceptor, negative regulator of TcpA | 0 | 0 | 0 | 0 | 0 | 0 |
